# Genome-shock deletion of a hybrid lethality gene breaks a reproductive barrier and facilitates speciation in *Nicotiana*

**DOI:** 10.3389/fpls.2025.1690873

**Published:** 2025-11-19

**Authors:** Shota Nagai, Kenji Kawaguchi, Hiroki Itakura, Kaho Matsumoto, Takahiro Iizuka, Kosaku Kobayashi, Kouki Nakata, Tetsuya Yamada, Wataru Marubashi, Masanori Yanase, Toshinobu Morikawa, Shuji Yokoi, Takahiro Tezuka

**Affiliations:** 1 Graduate School of Agriculture, Osaka Metropolitan University, Sakai, Osaka, Japan; 2 Research Institute of Environment, Agriculture and Fisheries, Habikino, Osaka, Japan; 3 Graduate School of Life and Environmental Sciences, Osaka Prefecture University, Sakai, Osaka, Japan; 4 School of Life and Environmental Sciences, Osaka Prefecture University, Sakai, Osaka, Japan; 5 United Graduate School of Agricultural Science, Tokyo University of Agriculture and Technology, Tokyo, Japan; 6 School of Agriculture, Meiji University, Kawasaki, Kanagawa, Japan; 7 Education and Research Field, School of Agriculture, Osaka Metropolitan University, Sakai, Osaka, Japan

**Keywords:** interspecific hybridization, hybrid lethality, reproductive isolation, genome shock, evolution, *Nicotiana*

## Abstract

Allopolyploidization plays an important role in the evolution of eukaryotes. Allopolyploids formed through interspecific hybridization and polyploidization undergo genetic and epigenetic changes in the early generations, known as ‘genome shock’. However, reproductive isolation often prevents interspecific hybridization. The mechanism by which a reproductively isolated species breaks reproductive barriers and crosses with other species is largely unknown, despite its importance in speciation and evolution. Here, we report the ultrahigh-frequency appearance of viable hybrids that overcame hybrid lethality, a type of reproductive isolation, in crosses between *Nicotiana tabacum* and *N*. *amplexicaulis*. Lethal hybrids exhibited Type II hybrid lethality characterized by browning of hypocotyl and roots at 28 °C, temperature sensitivity, and involvement of the Q chromosome from *N. tabacum* genome, indicating that *N. amplexicaulis* possesses the causal allele for hybrid lethality at the *Hybrid Lethality A1* (*HLA1*) locus. Random amplified polymorphic DNA, amplified fragment length polymorphism, and methylation-sensitive amplified polymorphism analyses have detected genetic and epigenetic changes in viable and lethal hybrids, suggesting the occurrence of genome shock during interspecific hybridization. We found that many viable hybrids exhibited *HLA1* locus deletion, indicating that it was the primary cause of overcoming hybrid lethality in these crosses. These findings demonstrate that genome shock-induced genetic changes promote the breakdown of reproductive barriers through the deletion of causal genes, providing insights into the mechanisms by which reproductively isolated species can overcome barriers and lead to the formation of new species.

## 1 Introduction

Allopolyploidization plays an important role in the evolution of eukaryotes (Rieseberg and Willis, 2007; Van de Peer et al., 2009). Allopolyploids formed through interspecific hybridization and polyploidization undergo genetic and epigenetic changes in early generations, as indicated by analyses of synthetic allopolyploids (Qiu et al., 2020). These changes, known as ‘genome shock’ (McClintock, 1984), have been widely studied in plant species such as rice (Wu et al., 2015), wheat (Shaked et al., 2001), rapeseed (Li et al., 2019), tobacco (Mhiri et al., 2019), and *Arabidopsis* (Madlung et al., 2002). However, reproductive isolation often prevents interspecific hybridization. If reproductive barriers are broken, new species can be established. Reproductive barriers are occasionally overcome; i.e., polyploidization, which balances homologous chromosome segregation and gene expression, has been reported as one mechanism for overcoming hybrid sterility, a type of reproductive barrier (Meeus et al., 2020; Mino et al., 2022; Ren et al., 2022). However, the mechanism by which reproductively isolated species break reproductive barriers and produce hybrid offspring remains largely unknown.

Reproductive isolation plays crucial roles in speciation and evolution. These barriers are divided into prezygotic and postzygotic barriers. Prezygotic isolation involves pollen–pistil incompatibility, conspecific pollen precedence, gametic incompatibility, and pistil-length mismatch (Rieseberg and Willis, 2007; Lee et al., 2008; Rieseberg and Blackman, 2010; Huang et al., 2023). Post-zygotic barriers include hybrid seed abortion, hybrid lethality, and hybrid sterility in the F_1_ generation, and hybrid breakdown in the F_2_ or later generations (Li J. et al., 2020; Dziasek et al., 2021; Xu et al., 2023). Hybrid lethality, which has been reported in many plant species, such as rice (Chen et al., 2014), wheat (Si et al., 2021), cotton (Deng et al., 2019), pepper (Shiragaki et al., 2020b), and *Arabidopsis thaliana* (Bomblies et al., 2007), is generally caused by the interaction between two dominant complementary genes from the parental plants (Bomblies and Weigel, 2007).

In the genus *Nicotiana*, many interspecific cross combinations result in hybrid lethality in seedlings. Hybrid lethality in this genus is classified into five types based on the early visible symptoms of hybrid seedlings: Type I, browning of the shoot apex and root tips; Type II, browning of the hypocotyl and roots; Type III, yellowing of true leaves; Type IV, formation of multiple shoots; and Type V, fading of shoot color (Yamada et al., 1999; Tezuka and Marubashi, 2012). Type II hybrid lethality occurs in most cross-combinations between the cultivated species *N. tabacum* and the wild species of the *Nicotiana* section *Suaveolentes*. Type II hybrid lethality is temperature-sensitive, observed at 28 °C but suppressed at elevated temperatures between 34 °C and 36 °C (Yamada et al., 1999; Tezuka et al., 2010), and is caused by the epistatic interaction between the dominant allele *Hla1–1* at the *HLA1* locus of *Suaveolentes* species and the dominant allele *Hla2–1* at the *HLA2* locus on the Q chromosome of *N. tabacum* (Iizuka et al., 2012; Ma et al., 2020; Nakata et al., 2021; Mino et al., 2022; He et al., 2023; Tezuka et al., 2024).

We previously reported that hybrid seedlings and plants that overcome Type II hybrid lethality and exhibit normal growth rarely appear spontaneously in the following cross combinations: *N. africana* × *N. tabacum* ‘Samsun NN’ (1.45% of hybrids), *N. tabacum* ‘Red Russian’ × *N. debneyi* (0.33%), *N. megalosiphon* × ‘Red Russian’ (0.60%), and *N. suaveolens* × ‘Red Russian’ (1.32%) (Tezuka and Marubashi, 2006b; Tezuka et al., 2010). Tezuka et al. (2012) reported that partial deletion of the Q chromosome, where the *HLA2* locus is located, is responsible for the occurrence of viable hybrids in the *N. tabacum* × *N. africana* cross. Other authors have also reported that similar deletions in the terminal region of the Q chromosome are thought to result in viable hybrids in the *N. tabacum* ‘TN 90LC’ × *N. africana* (0.12%) and *N. suaveolens* × *N. tabacum* ‘Hicks-2’ (0.09%) crosses (Hancock et al., 2015; Nakata et al., 2021). One reason for these deletions is the reciprocal translocation between homoeologous chromosomes during pollen formation in the allotetraploid *N. tabacum* (Nakata et al., 2021). Despite these findings, the mechanism of overcoming hybrid lethality in interspecific crosses through allopolyploidization remains unknown.

In this study, we report that crosses between *N. amplexicaulis* (section *Suaveolentes*) and *N. tabacum* yield significantly more viable hybrids that overcome hybrid lethality than other lethal crosses. We characterized hybrid lethality based on phenotypic symptoms, temperature sensitivity, and the *N. tabacum* chromosome responsible for the hybrid lethality. To clarify the mechanism of the ultrahigh-frequency appearance of viable hybrids, we investigated the genetic changes in hybrids using random amplified polymorphic DNA (RAPD) and amplified fragment length polymorphism (AFLP) analyses and the epigenetic changes in hybrids using methylation-sensitive amplified polymorphism (MSAP) analysis. In addition, we analyzed the genetic changes or alterations in the expression of the two causal genes for hybrid lethality. Based on these results, we discuss the effects of breaking reproductive isolation on natural plant evolution.

## 2 Materials and methods

### 2.1 Plant materials

Two cultivars of *N. tabacum*, ‘Red Russian’ and ‘Samsun NN,’ and three accessions of *N. amplexicaulis*, JT, PI 271989, and PI 555682, were used in this study. We also used F_1_ monosomic progeny from the cross between *N. tabacum* Q chromosome monosomic line Haplo-Q and disomic ‘Samsun NN’ (Tezuka et al., 2004). All plants were cultivated in a greenhouse under natural day lengths and used for crossing experiments.

### 2.2 Interspecific crosses and investigation of lethal symptoms in hybrid seedlings

In conventional crossing, the flowers of plants used as the maternal parent are emasculated one day before anthesis and pollinated with pollen from paternal parent plants. The obtained seeds were treated with a 0.5% gibberellic acid (GA_3_) solution for 30 minutes, soaked in a 5% sodium hypochlorite solution for 15 minutes, and rinsed three times with sterile water. The sterilized seeds were sown in Petri dishes containing 1/2 MS medium (Murashige and Skoog, 1962) supplemented with 1% sucrose and solidified with 0.2% Gelrite (pH 5.8) and then cultured at 28 °C under continuous illumination (approximately 150 μmol m^−2^ s^−1^).

Test-tube pollination and ovule culture were conducted as previously described (Tezuka and Marubashi, 2004). Hybrid seedlings obtained from the cross between ‘Samsun NN’ and *N. amplexicaulis* JT by the test-tube pollination and ovule culture were transferred to flat-bottomed test tubes (35 mm diameter, 120 mm length) containing 20 mL of 1/2 MS medium supplemented with 1% sucrose and solidified with 0.2% Gelrite (pH 5.8) immediately after germination and cultured at 28 °C under continuous illumination.

Hybrid seedlings obtained from conventional crossing and test-tube pollination with ovule culture were observed for lethal symptoms at 28 °C. To investigate temperature sensitivity of hybrid lethality, 20 hybrid seedlings from the cross *N. amplexicaulis* JT and ‘Red Russian’ were transferred into flat-bottomed test tubes (25 mm diameter, 100 mm) containing 10 mL of 1/2 MS medium supplemented with 1% sucrose and solidified with 0.2% Gelrite (pH 5.8) immediately after germination and were cultured at 34 °C. Hybrid seedlings cultured at 34 °C for 30 d after germination (DAG) were transferred to 28 °C under continuous illumination. Hybrid seedlings that survived for more than 30 d after transfer were potted and cultivated in a greenhouse under natural day lengths.

### 2.3 Investigation of *N. tabacum* Q-chromosome involvement in hybrid lethality

To investigate the involvement of the *N. tabacum* Q chromosome in hybrid lethality, hybrid seedlings from a cross between the F_1_ monosomic progeny of Haplo-Q × ‘Samsun NN’ and *N. amplexicaulis* JT were obtained by test-tube pollination with ovule culture. The hybrid seedlings were transferred to flat-bottomed test tubes (30 mm diameter, 120 mm length) containing 25 mL of 1/2 MS medium supplemented with 1% sucrose and solidified with 0.2% Gelrite (pH 5.8) immediately after germination and cultured at 34 °C under continuous illumination. Seedlings were subcultured in fresh medium every 3 weeks. After analysis using Q-chromosome-specific DNA markers, seedlings were transferred to 28 °C under continuous illumination.

### 2.4 PCR analyses

Total DNA was extracted from young leaves of each plant using a cetyltrimethylammonium bromide (CTAB)-based method (Murray and Thompson, 1980). The Q-chromosome-specific DNA markers QCS2, QCS3, and QCS4 were detected as previously described (Tezuka et al., 2004; Tezuka and Marubashi, 2006a). To investigate the *HLA2* locus, the SSR markers PT30137, PT30342, and PT30365 (Bindler et al., 2011, 2007), were used. *HLA2*-specific sequence tagged site (STS) marker HLA2-STS was developed based on the nucleotide sequence of the *Hla2–1* allele (Ma et al., 2020) (**Supplementary Table 1**). To investigate the *HLA1* locus, three CAPS markers linked to *HLA1*, Nb14-CAPS, NbRGH1-CAPS, and Nb49-CAPS (Tezuka et al., 2021, 2024) were used. However, Nb14-CAPS and Nb49-CAPS were used as STS markers (Nb14-STS and Nb49-STS) because polymorphisms were detected between the species used in this study without restriction enzyme treatment. Because NbRGH1-CAPS treated with restriction enzyme *Bsr*I showed no polymorphism between ‘Samsun NN’ and *N. amplexicaulis*, PCR products of NbRGH1-CAPS from both were completely sequenced. Subsequent digestion with restriction enzyme *Eco*T22I revealed a polymorphism (NbRGH1-*Eco*T22I). Five new STS markers, Nb138-STS, Nb88-STS, Nb89-STS, Nb93-STS, and Nb135-STS, were developed based on the scaffold sequence Nbe.v1.1.chr15 in *N. benthamiana* genome Nbe.v1.1. These markers were detected by PCR, using the same method as previously described (Tezuka et al., 2021).

### 2.5 RAPD, AFLP, and MSAP analysis

RAPD analysis was performed for viable hybrid seedlings using 20 random 10-mer oligonucleotide primers (Kit A; Operon Technologies, Inc., Alameda, CA, USA), as previously described (Tezuka et al., 2007). Only clear bands were observed.

For AFLP and MSAP analysis, hybrid seedlings obtained from the cross between ‘Samsun NN’ and *N. amplexicaulis* JT were transferred into flat-bottomed test tubes (35 mm diameter, 120 mm) containing 20 mL of 1/2 MS medium supplemented with 1% sucrose and solidified with 0.2% Gelrite (pH 5.8) immediately after germination at 28 °C and were cultured at 34 °C to suppress hybrid lethality. After extracting DNA for AFLP and MSAP analyses using the CTAB-based method (Murray and Thompson, 1980), the seedlings were transferred to 28 °C under continuous illumination and classified into viable and lethal hybrids based on their lethal phenotypes.

AFLP analysis was conducted as previously described by Vos et al. (1995), with a few modifications. Total DNA was double-digested with *Eco*RI and *Mse*I and ligated to *Eco*RI and *Mse*I adapters. The pre-amplification reaction was performed using *Eco*RI+A (E01) and *Mse*I+C (M02) primers. The PCR products were then diluted tenfold with sterile water and used as templates for the second amplification reaction. Primers with three selective nucleotides, 7 *Eco*RI+ANN (N indicates A, T, G, or C; E37 to E43) and 16 *Mse*I+CNN (M47 to M62) primers, were used for the second amplification reaction with 112 combinations (**Supplementary Table 2**). In both amplification reactions, KAPA Taq EXtra DNA polymerase (Kapa Biosystems, Wilmington, MA, USA) was used, and PCR amplification was performed using a PC-818A Program Temp Control System (Astec, Fukuoka, Japan). The second PCR product was separated by electrophoresis on an 8% polyacrylamide gel and visualized by staining with ethidium bromide. Only bands smaller than 800 bp were identified. Each band was named according to the primer combination, and the subsequent number indicated the estimated DNA fragment size.

MSAP analysis was conducted as previously described by Fulneček and Kovařík (2014), with a few modifications, using the same DNA used for AFLP analysis. Total DNA was double-digested with *Eco*RI and *Hpa*II or *Msp*I and ligated to *Eco*RI adapter and *Hpa*II or *Msp*I adapters. The isoschizomer pairs, *Hpa*II and *Msp*I, are methylation-sensitive restriction enzymes that cleave the CCGG sequence. *Hpa*II can only cleave nonmethylated CCGG sequences or those hemi-methylated at the external cytosine. *Msp*I can cleave non-methylated CCGG sequences and those hemi- or fully methylated at the internal cytosines. However, it cannot cleave CCGG sequences that are hemi-methylated or fully methylated at the external cytosine (Reyna-López et al., 1997). The preamplification reaction was performed using *Eco*RI+A (E01) and *Hpa*II+T or *Msp*I+T (HM01) primers. The PCR products were then diluted tenfold with sterile water and used as templates for the second amplification reaction. Primers with three selective nucleotides, 16 *Eco*RI+ANN (N indicates A, T, G, or C; E37–E46), and 4 *Hpa*II+TAN or *Msp*I+TAN (HM47–HM50) primers were used for the second amplification reaction with 64 combinations (**Supplementary Table 2**). Both amplification reactions and band detection were carried out as described for the AFLP analysis.

### 2.6 RT-PCR analysis of *Hla2–1* expression

For RT-PCR analysis, hybrid seedlings obtained from the cross between ‘Samsun NN’ and *N. amplexicaulis* JT were cultured at 28 °C *in vitro*. At 4 DAG, the phenotype of each hybrid was evaluated and classified as either viable or lethal. Subsequently, before the lethal hybrids died, viable and lethal hybrids were transferred to flat-bottomed test tubes (35 mm diameter, 120 mm length) containing 20 mL of 1/2 MS medium supplemented with 1% sucrose, solidified with 0.2% Gelrite (pH 5.8), and cultured at 34 °C under continuous illumination to increase the growth of the hybrids. At 14 DAG, the seedlings were transferred to 28 °C under continuous illumination to induce hybrid lethality. One day after transfer, total RNA was extracted from the whole plant using TRIzol reagent (Invitrogen Inc., Carlsbad, USA) and then treated with RNase-free DNase (Promega Co., Madison, WI, USA) according to the manufacturer’s protocol. First-strand cDNA was synthesized from 1 μg total RNA using oligo (dT)_18_ primers and ReverTra Ace (Toyobo Co., Ltd., Osaka, Japan).

To analyze *Hla2–1* expression, primers for the *HLA2*-specific STS marker Nt6549g30-1 (Nakata et al., 2021) were used. Primers for *Actin* were designed by Shiragaki et al. (2020a). PCR reaction mixtures consisted of 1 × KAPA Taq EXtra Buffer (Kapa Biosystems, Wilmington, MA, USA), 0.2 mM each dNTP, 0.2 µM each primer, 0.25 U KAPA Taq EXtra DNA polymerase (Kapa Biosystems, Wilmington, MA, USA), and 1 µL of diluted cDNA (first-strand cDNA: nuclease-free water = 1:4) in a total volume of 10 µL. PCR amplification was performed using a PC-818A Program Temp Control System (Astec, Fukuoka, Japan) programmed for 3 min at 94 °C for initial denaturation, followed by 30 cycles of denaturation at 94 °C for 30 s, annealing at 55 °C for 30 s, and extension at 72 °C for 60 s, with a final 5-min extension at 72 °C. The PCR products were separated by electrophoresis on 3.0% agarose gels in TBE buffer and visualized by staining with ethidium bromide.

## 3 Results

### 3.1 Hybrid lethality observed in hybrid seedlings between *N. amplexicaulis* and *N. tabacum*

**Figure 1:**
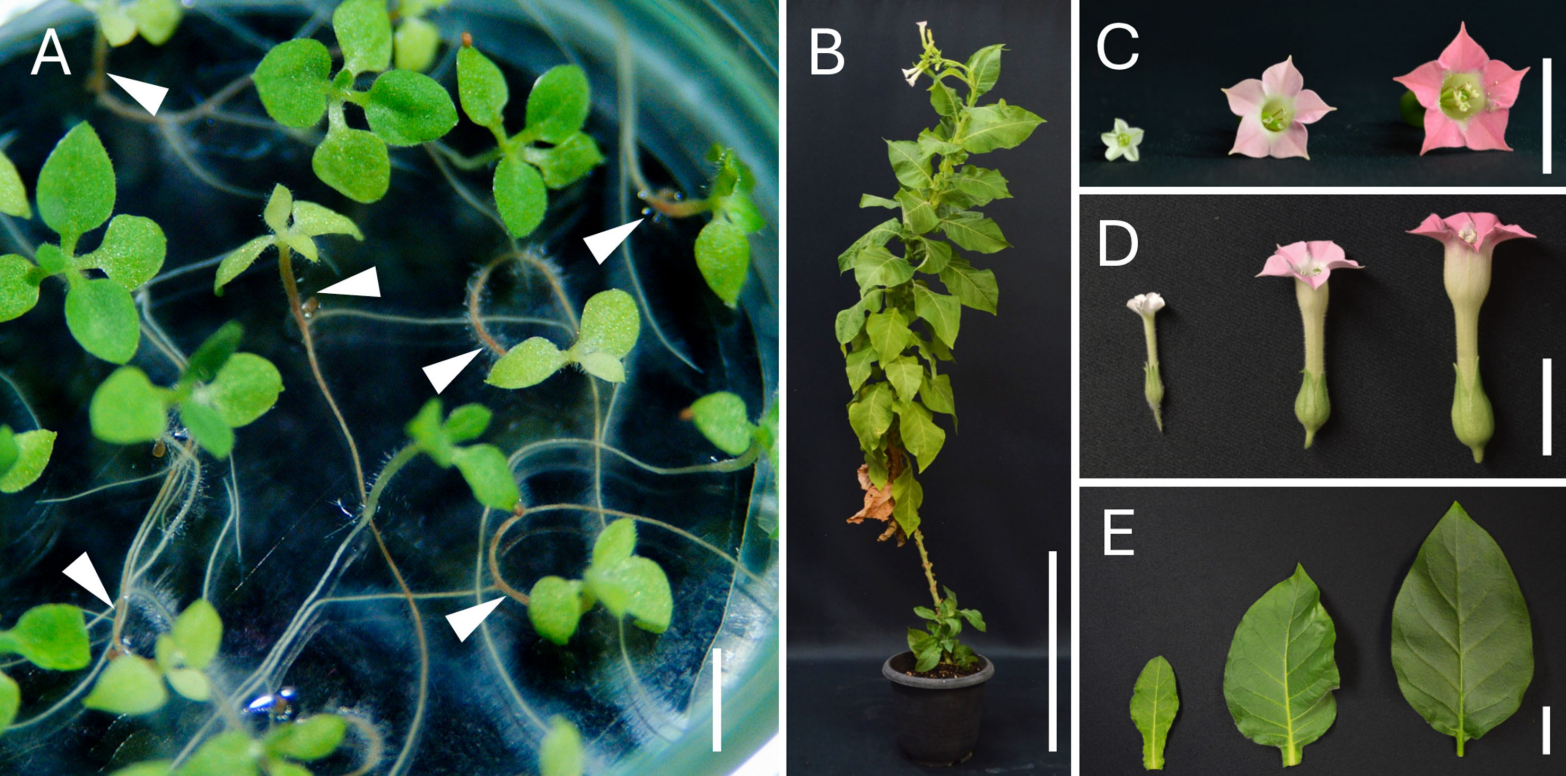
Hybrid seedlings and plants from the cross *N. amplexicaulis* JT × *N. tabacum.***(A)** Characteristic early symptoms of hybrid lethality in the hybrid seedlings from the cross *N. amplexicaulis* × ‘Red Russian’. Arrowheads indicate browning of hypocotyl and roots, which is characteristic of type II hybrid lethality. **(B)** Shape of a viable hybrid plant from the cross *N. amplexicaulis* × ‘Samsun NN’ that has grown to maturity and flowered. **(C, D)** Flowers of *N. amplexicaulis*, a viable hybrid plant, and ‘Samsun NN’ (left to right). **(E)** Leaves of *N. amplexicaulis*, a viable hybrid plant, and ‘Samsun NN’ (left to right). Scale bars = 0.5 cm **(A)**, 30 cm **(B)**, and 2 cm **(C-E)**.

Three accessions of *N. amplexicaulis* (JT, PI 271989, and PI 555682) were crossed with *N. tabacum*. First, *N. amplexicaulis* JT was reciprocally crossed with two cultivars of *N. tabacum*, ‘Red Russian’ and ‘Samsun NN’. Seeds were only obtained in the crosses using *N. tabacum* as the male parent, and no capsules were produced from crosses using *N. tabacum* as the female parent, suggesting that fertilization was unsuccessful (**Table 1**). After sowing the seeds *in vitro* at 28 °C, 179 and 116 hybrid seedlings were obtained from crosses using ‘Red Russian’ and ‘Samsun NN’ as male parents, respectively. Of these, 159 and all 116 seedlings from each cross were subsequently cultured at 28 °C. Regardless of *N. tabacum* cultivar, more than half of the hybrid seedlings showed normal growth, whereas the remaining hybrid seedlings exhibited Type II hybrid lethality, characterized by early symptoms of hypocotyl and root browning (**Table 2**; **Figure 1A**). Based on these results, *N. amplexicaulis* PI 271989 and PI 555682 were crossed with ‘Red Russian’ as the male parent. PI 271989 yielded 17 viable hybrid seedlings (29.3%) and 41 lethal hybrid seedlings (70.7%) at 28 °C. Similarly, PI 555682 yielded five viable hybrid seedlings (26.3%) and 14 lethal hybrid seedlings (73.7%) at 28 °C (**Table 1**). The lethality of the hybrid seedlings from the two crosses was Type II. Viable hybrid seedlings from all four crosses were potted and cultivated in a greenhouse. All hybrids matured and flowered without any lethality symptoms. The morphological characteristics of the hybrid plants were uniform for each cross combination. The leaf shape, flower shape, and flower color of the hybrid plants were intermediate in appearance between those of the parents in all cross combinations (**Figures 1B–E**). Since the hybrid lethality observed in the four crosses appeared to be due to the same mechanism, subsequent studies focused on crosses between *N. amplexicaulis* JT and *N. tabacum* cultivars (‘Red Russian’ and/or ‘Samsun NN’).

**Table 1:** Conventional crossings between three *N. amplexicaulis* accessions and *N. tabacum*.

Cross combination	No. of flowers pollinated	No. of capsules obtained	No. of seeds sown	No. of hybrids obtained (%)
*N. amplexicaulis* JT × ‘Red Russian’	20	8	198	179 (90.4)
‘Red Russian’ × *N. amplexicaulis* JT	20	0	-	-
*N. amplexicaulis* JT × ‘Samsun NN’	20	13	117	116 (99.1)
‘Samsun NN’ × *N. amplexicaulis* JT	20	0	-	-
*N. amplexicaulis* PI 271989 × ‘Red Russian’	6	1	58	58 (100)
*N. amplexicaulis* PI 555682 × ‘Red Russian’	9	4	20	19 (95.0)

**Table 2:** Viability of hybrid seedlings between three *N. amplexicaulis* accessions and *N. tabacum* at 28 °C or 34 °C.

Cross combination	Culture temperature	No. of hybrids			p^b^
		Total	Viable (%)	Lethal (%)	
*N. amplexicaulis* JT × ‘Red Russian’	28 °C	159	83 (52.2)	76 (47.8)	7.34 × 10^-1^
	34 °C	20^a^	20 (100)	0 (0)	–
	34 °C→28 °C	20^a^	10 (50.0)	10 (50.0)	1.00
*N. amplexicaulis* JT × ‘Samsun NN’	28 °C	116	69 (59.5)	47 (40.5)	1.87 × 10^-1^
‘Samsun NN’ × *N. amplexicaulis* JT	28 °C	113	15 (13.3)	98 (86.7)	2.34 × 10^-9^
*N. amplexicaulis* PI 271989 × ‘Red Russian’	28 °C	58	17 (29.3)	41 (70.7)	3.62 × 10^-2^
*N. amplexicaulis* PI 555682 × ‘Red Russian’	28 °C	19	5 (26.3)	14 (73.7)	1.91 × 10^-1^

a The same 20 hybrids were used.

b Fisher’s exact test (two-sided) for deviation from expected 1:1 ratio (viable:lethal).

To obtain hybrid seedlings of *N. tabacum* with *N. amplexicaulis* JT as the male parent, we performed test-tube pollination and ovule culture, as combined techniques are useful for bypassing the prezygotic barriers (Tezuka et al., 2010). Fifty-five placentas of *N. tabacum* ‘Samsun NN’ were pollinated with *N. amplexicaulis* JT pollen *in vitro*, resulting in 790 enlarged ovules. These ovules were cultured with fresh media at 28 °C, and 113 hybrid seedlings were obtained. Of these, 98 seedlings (86.7%) showed type II hybrid lethality and 15 seedlings (13.3%) showed normal growth (**Table 2**).

### 3.2 Effect of an elevated temperature on hybrid lethality

Hybrid lethality in crosses between species of section *Suaveolentes* and *N. tabacum* is suppressed at elevated temperatures (32 °C –36 °C) (Mino et al., 2002; Yamada and Marubashi, 2003; Tezuka et al., 2007, 2010). We investigated whether hybrid lethality in the cross between *N. amplexicaulis* JT and *N. tabacum* ‘Red Russian’ could also be suppressed under elevated temperature conditions. When the remaining 20 of 179 hybrid seedlings obtained by *in vitro* sowing were transferred to 34 °C, all of them exhibited normal growth without lethality until at least 30 DAG (**Table 2**). These hybrid seedlings were transferred to 28 °C at 30 DAG to assess whether lethality could be suppressed only during culture at elevated temperatures. Ten hybrid seedlings (50%) exhibited hybrid lethality and died, whereas the remaining 10 (50%) continued to grow.

### 3.3 Involvement of the *N. tabacum* Q chromosome in hybrid lethality

To investigate whether the Q chromosome of *N. tabacum* is responsible for hybrid lethality, monosomic analysis was conducted using monosomic plants lacking one copy of the Q chromosome, which can be easily identified from F_1_ progeny from the cross between *N. tabacum* Q chromosome monosomic line Haplo-Q and ‘Samsun NN’ using DNA markers (Tezuka et al., 2004). In the analysis, although test-tube pollination and ovule culture were required, monosomic plants were crossed with *N. amplexicaulis* as male parents because the probability of transmission of the monosomic chromosome through pollen was remarkably low (Olmo, 1935). Sixteen placentas of Q chromosome monosomic plants were pollinated with *N. amplexicaulis* JT pollen, resulting in 327 enlarged ovules. After ovule culture at 28 °C, 13 hybrid seedlings were obtained and transferred to 34 °C to suppress hybrid lethality. Hybrid seedlings were assessed for the presence or absence of the Q chromosome using two Q-chromosome-specific (STS) markers: QCS2 and QCS3 (Tezuka et al., 2004; Tezuka and Marubashi, 2006b). Both markers were detected in six hybrid seedlings, but neither marker was detected in seven hybrid seedlings. When these hybrid seedlings were transferred from 34 °C to 28 °C, all seedlings possessing the Q chromosome showed hybrid lethality and died, whereas all seedlings lacking the Q chromosome survived without lethal symptoms (**Table 3**). These results suggest that hybrid lethality was caused by the epistatic interaction between the *Hla1–1* allele at the *HLA1* locus in *N. amplexicaulis* and the *Hla2–1* allele at the *HLA2* locus on the Q chromosome in *N. tabacum*, similar to the Type II hybrid lethality observed in crosses between other *Suaveolentes* species and *N. tabacum*.

**Table 3:** Relationship between the Q chromosome and hybrid lethality observed in the cross between *N. tabacum* and *N. amplexicaulis* JT.

Cross combination	DNA markers*	No. of hybrids		
		Total	Viable	Lethal
(Haplo-Q × ‘Samsun NN’) × *N. amplexicaulis*	+	6	0	6
	−	7	7	0

* ‘+’ indicates that Q-chromosome-specific STS markers, QCS2 and QCS3, were detected and ‘−’ indicates that they were not.

### 3.4 Involvement of *N. amplexicaulis* heterozygosity in viable hybrid formation

The occurrence rates of viable hybrids resulting from crosses between *N. amplexicaulis* accessions and *N. tabacum* were high (**Table 1**). Among the parental species used in this study, although *N. tabacum* cultivars were highly inbred, the homozygosity and heterozygosity of *N. amplexicaulis* remain unclear. Considering the results that the ratio of viable hybrids to lethal hybrids was close to 1:1 (Fisher’s exact test, two-sided, p > 0.05) in crosses between *N. amplexicaulis* JT and *N. tabacum* cultivars (**Table 2**), the heterozygosity of the *HLA1* locus might be responsible for the occurrence of viable hybrids. To test this hypothesis, we newly prepared four plants of *N. amplexicaulis* JT as the first generation (S_0_) and their self-progenies (S_1_ generation; ten plants each from the four S_0_ plants) and crossed them with *N. tabacum* ‘Red Russian’ as the male parent. If the frequent occurrence of viable hybrids was due to the heterozygosity of *HLA1*, a quarter of the S_1_ plants (homozygous for the recessive *hla1–2* allele) would produce only viable hybrids, half of the S_1_ plants (heterozygous) would produce viable and lethal hybrids in a 1:1 ratio, and the remaining quarter of the S_1_ plants (homozygous for *Hla1–1* allele) would produce only lethal hybrids. After crosses with *N. tabacum*, four S_0_ plants produced viable hybrids at percentages of 8.9%–63.2%. All S_1_ plants produced viable hybrids with occurrence rates ranging from 3.2% to 49.2%; however, the rates varied among plants within each lineage, ruling out the possibility that the *HLA1* locus was heterozygous (**Supplementary Table 3**).

### 3.5 Genetic changes in hybrid seedlings detected by RAPD and AFLP analyses

**Figure 2:**
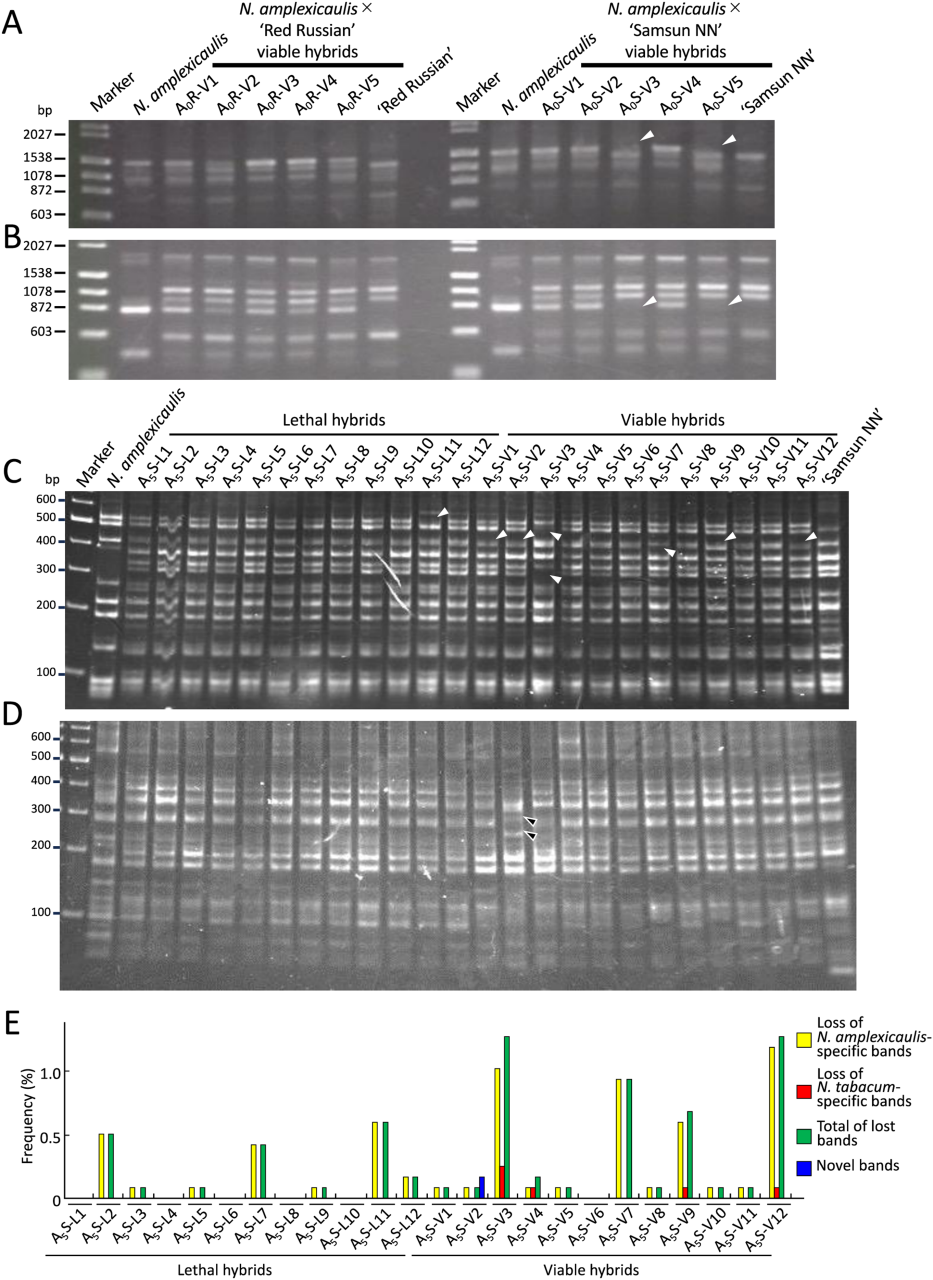
Genetic changes in hybrid plants identified by RAPD and AFLP analysis. **(A, B)** Loss of bands identified in hybrid plants by RAPD analysis using primers OPA-05 **(A)** and OPA-09 **(B)**. White arrowheads indicate the disappeared bands. Marker lane, DNA size markers (λ/Hind III and φX174/Hae III). **(C)** Lost bands identified in hybrid plants by AFLP analysis using E40-M55 primer pair. White arrowheads indicate the disappeared bands. Marker lane, DNA size marker (GeneRuler DNA ladder mix). **(D)** Nobel bands identified in hybrid plants by AFLP analysis using E39-M49 primer pair. Black arrowheads indicate novel bands. Marker lane, DNA size marker (GeneRuler DNA ladder mix). **(E)** Frequency of lost or novel bands in each hybrid identified by AFLP analysis.

To investigate DNA mutations in viable hybrids, RAPD analysis was performed on five viable hybrids from the cross between S_0_ plants and *N. tabacum* ‘Red Russian’ (A_0_R-V1–5) and five viable hybrids from the cross between S_0_ plants and *N. tabacum* ‘Samsun NN’ (A_0_S-V1–5). Using 20 OPA primers, we detected 19 N*. amplexicaulis*-specific bands, 33 N*. tabacum* (‘Red Russian’ and ‘Samsun NN’) specific bands, and 24 common bands between the parents. No polymorphism was detected between ‘Red Russian’ and ‘Samsun NN’. In the two viable hybrids, A_0_S-V3 and A_0_S-V5, two *N. amplexicaulis*-specific bands detected using primers OPA-05 or OPA-19 were absent, suggesting that genetic changes occurred in the hybrid seedlings (**Figures 2A, B**; **Table 2**).

To investigate genetic changes more comprehensively, we conducted AFLP analysis using 112 primer sets on 12 viable hybrids (A_5_S-V1–12) and 12 lethal hybrids (A_5_S-L1–12) obtained from the cross between an *N. amplexicaulis* JT S5 plant and ‘Samsun NN’. AFLP analysis detected 224 N*. amplexicaulis*-specific bands, 323 N*. tabacum*-specific bands, and 588 common bands between the parents. Similar to RAPD analysis, no polymorphism was detected between ‘Red Russian’ and ‘Samsun NN’. Of the bands detected, 39 N*. amplexicaulis*-specific bands and four *N. tabacum*-specific bands were absent in 11 viable hybrids and seven lethal hybrids (**Figures 2C, E**; **Supplementary Table 4**). The number of absent bands varied depending on hybrids. Excluding six hybrids with no missing bands, the frequencies of lost bands in each hybrid ranged from 0.09% (one band) to 1.32% (15 bands) (**Figure 2E**). Among the lost bands, some were absent in only one hybrid, whereas others were absent in up to four hybrids (**Supplementary Table 4**). No bands were consistently missing across all the viable or lethal hybrids. In addition, two novel bands that were not present in the parents were observed in the viable hybrid A_5_S-V2 (**Figures 2D, E**).

### 3.6 Epigenetic changes in hybrid seedlings detected by MSAP analysis

**Figure 3:**
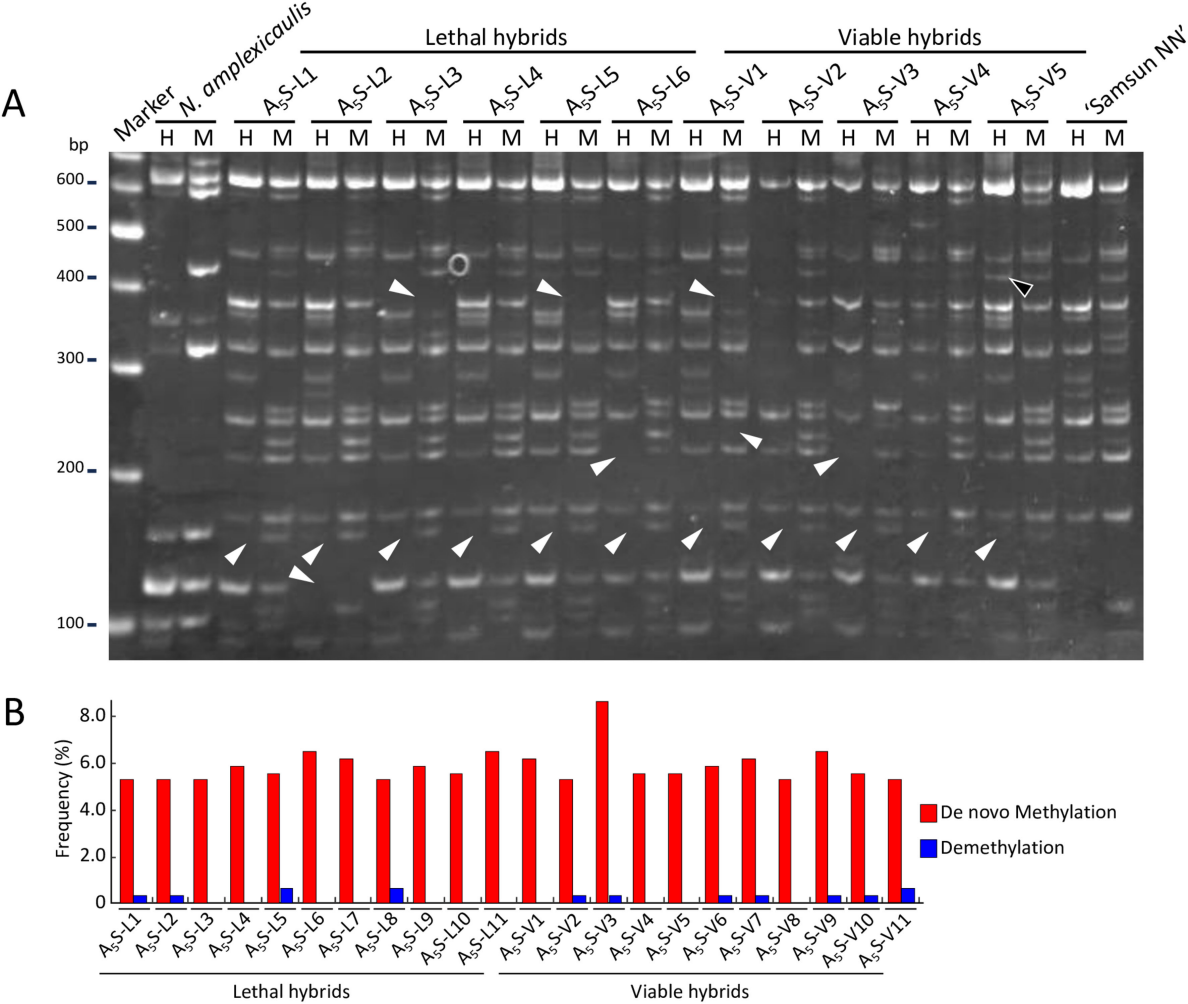
Epigenetic changes in hybrid plants identified by MSAP analysis. **(A)** An example of bands indicating variation of DNA methylation with the E35-HM49 primer pair. Lane H, PCR products obtained by digestion with *Eco*RI–*Hpa*II; lane M, PCR products obtained by digestion with *Eco*RI–*Msp*I; Marker lane, DNA size marker (GeneRuler DNA ladder mix). White arrowheads indicate MSAP bands showing *de novo* methylation, and black arrowheads indicate MSAP bands showing demethylation. **(B)** Frequency of methylation changes in each hybrid identified by MSAP analysis.

To investigate whether methylation changes were involved in the occurrence of viable hybrids, we conducted MSAP analysis using *Eco*RI in combination with the isoschizomer pair *Hpa*II and *Msp*I, which allows for genome-wide determination of DNA methylation status by comparing band patterns generated using a pair of isoschizomer restriction enzymes with different sensitivities to methylation (Reyna-López et al., 1997). After MSAP analysis using 64 primer sets on 11 viable hybrids (A_5_S-V1–11) and 11 lethal hybrids (A_5_S-L1–11), which were also used for AFLP analysis, a total of 324 MSAP loci were detected, with 40 loci indicating *de novo* methylation and four loci indicating demethylation in hybrids compared to their parents (**Figure 3A**). The frequencies of *de novo* methylation and demethylation in the hybrids ranged from 5.25% to 8.64% and from 0% to 0.62% of the total MSAP loci, respectively (**Figure 3B**). Among the 44 loci showing methylation changes, 16 were consistently detected in all the hybrids. For the remaining 28 loci, the number of hybrids with methylation changes varied depending on the locus. The methylation changes most frequently observed in viable hybrids were detected in seven of the 11 viable hybrids, but this change was also detected in five of the 11 lethal hybrids. No methylation change was consistently detected across all viable or lethal hybrids.

### 3.7 Investigation of loci causing hybrid lethality

**Figure 4:**
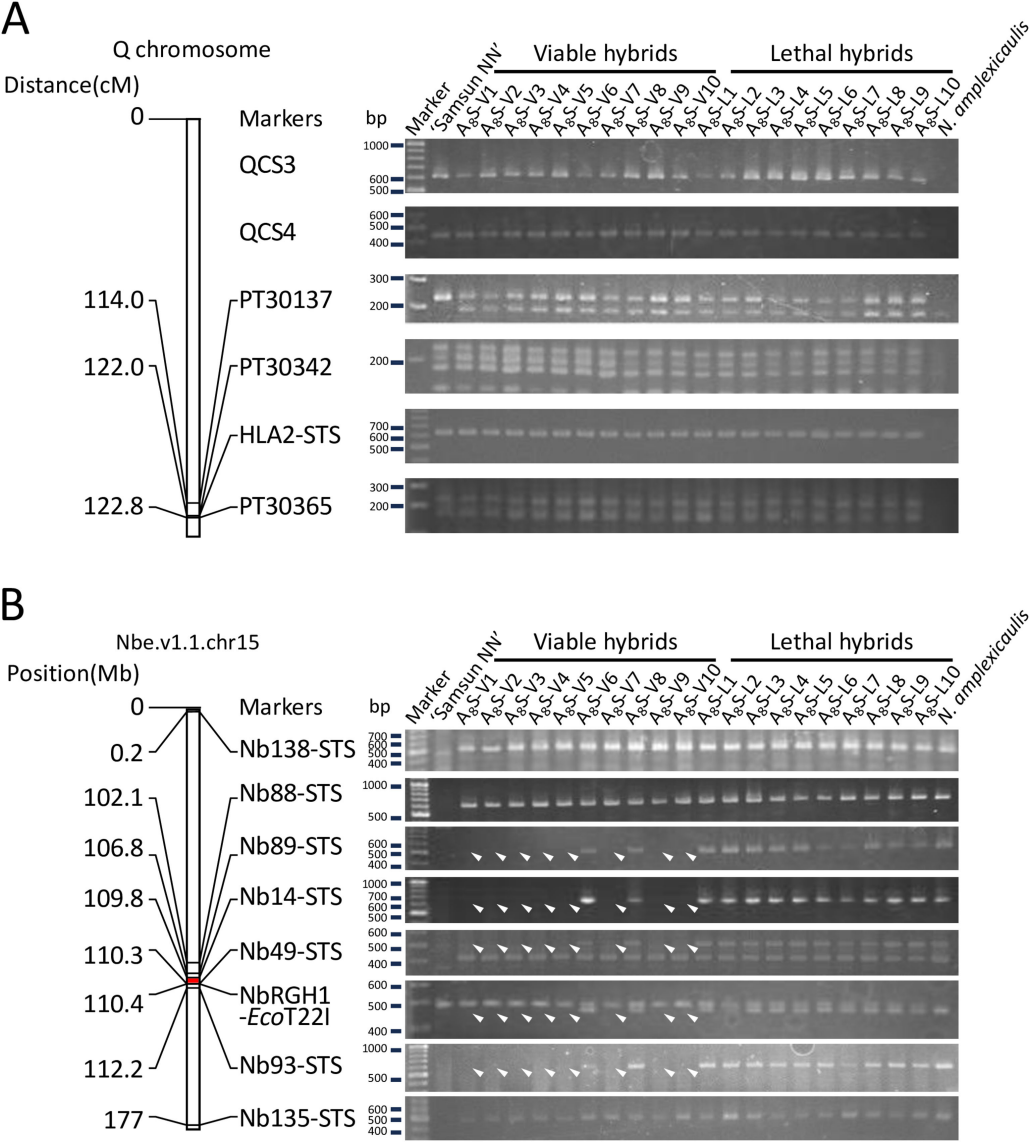
PCR analysis of the two hybrid lethality loci *HLA2***(A)** and *HLA1***(B)**. **(A)** Linkage map showing marker positions on the Q chromosome involving *HLA2* and the band patterns detected with the markers. **(B)** Physical map showing marker positions in Nbe.v1.1.chr15 scaffold, which involves *HLA1*, from the Nbe.v1.1 genome of *N. benthamiana*, and the band patterns detected with the markers. Position (Mb) indicates the approximate position of the markers in Nbe.v1.1.chr15 scaffold. The Red region indicates the region containing the *HLA1* locus. Arrowheads indicate the disappeared bands. Marker lane, size marker (GeneRuler DNA ladder mix).

Possible mutations or losses of *Hla1–1* and *Hla2–1* alleles were investigated in 10 viable (A_8_S-V1–10) and 10 lethal hybrids (A_8_S-L1–10) obtained from a cross between an S_8_ plant and *N. tabacum* ‘Samsun NN’. The *N. tabacum HLA2* locus is closely linked to the SSR marker PT30342 on the Q chromosome (*N. tabacum* linkage group 11) (Ma, 2017; Bindler et al., 2007; Tezuka et al., 2012). We investigated the presence of three SSR markers linked to *HLA2*, including PT30342 (Bindler et al., 2007, 2011), and two unmapped Q chromosome-specific DNA markers, QCS3 and QCS4, (Tezuka et al., 2004; Tezuka and Marubashi, 2006a), in the hybrids. Additionally, an STS marker, HLA2-STS, which was newly developed based on the sequence of *HLA2*, was also investigated (**Supplementary Table 1**). All markers were detected in both the viable and lethal hybrids (**Figure 4A**).

Another hybrid lethality gene *HLA1* was mapped between two cleaved amplified polymorphic sequence (CAPS) markers, NbRGH1-CAPS and Nb14-CAPS, located on Nbe.v1.1.chr15 in *N. benthamiana* genome Nbe.v1.1 (Kurotani et al., 2023; Tezuka et al., 2021). In addition to these markers, we used the STS marker Nb49 located between NbRGH1-CAPS and Nb14-CAPS (Tezuka et al., 2024), and five other STS markers, Nb138-STS, Nb88-STS, Nb89-STS, Nb93-STS, and Nb135-STS, which were newly developed based on the scaffold sequence (**Supplementary Table 1**). Five markers from Nb89-STS to Nb93-STS in the physical map were detected in all lethal hybrids but not in eight of the 10 viable hybrids (**Figure 4B**). In contrast, markers located outside this region (Nb138-STS and Nb88-STS on the same side, and Nb135-STS on the other side) were detected in all hybrids.

Additionally, to investigate whether suppression of *Hla2–1* expression contributed to the suppression of hybrid lethality, we conducted RT-PCR analysis. Because the 10 viable hybrids (A_8_S-V1–10) and 10 lethal hybrids (A_8_S-L1–10) used in the above marker analysis had died due to natural aging and hybrid lethality, respectively, 21 viable (A_8_S-V11–31) and three lethal (A_8_S-L11–13) hybrids were newly obtained from the cross between the S_8_ plant and ‘Samsun NN’, and used for the gene expression analysis. *Hla2–1* was expressed in all the hybrids (**Supplementary Figure 1**).

## 4 Discussion

### 4.1 Characterization of hybrid lethality in crosses between *N. amplexicaulis* and *N. tabacum*

Hybrid lethality in reciprocal crosses between *N. amplexicaulis* and *N. tabacum* was characterized by lethal symptoms, such as browning of the hypocotyls and roots. In addition, hybrid lethality in the cross *N. amplexicaulis* × *N. tabacum* was temperature sensitive, and the Q chromosome in *N. tabacum* genome was involved in hybrid lethality. These characteristics are consistent with Type II hybrid lethality caused by epistatic interactions between the *Hla1–1* allele from *Suaveolentes* species and the *Hla2–1* allele from *N. tabacum* (Iizuka et al., 2012; Ma et al., 2020; Tezuka et al., 2024). Considering that species in the monophyletic section *Suaveolentes* are closely related (Chase et al., 2003; Clarkson et al., 2004; D’Andrea et al., 2023), the present results indicate that *N. amplexicaulis* also carries the *Hla1–1* allele. This is also supported by a report by Ma et al. (2020), where all hybrids survived after crossing *N. amplexicaulis* with *N. tabacum* mutant for the *HLA2* gene.

Crosses between *N. amplexicaulis* and *N. tabacum* produced a large number of normal hybrids compared to crosses using other *Suaveolentes* species. Previous studies have reported the occurrence of viable hybrids at extremely low frequencies in crosses between *Suaveolentes* and *N. tabacum* (Tezuka et al., 2010; Tezuka and Marubashi, 2006b; Hancock et al., 2015; He et al., 2019; Nakata et al., 2021) (**Supplementary Table 5**). In contrast, the present study demonstrated an ultrahigh frequency of viable hybrids, ranging from 3.2% to 63.2% (**Table 2** and **Supplementary Table 3**). To date, such a high frequency of viable hybrids has not been reported in other plant species where hybrid lethality has been observed.

### 4.2 Causes of ultrahigh frequency appearance of viable hybrids

It is important to understand the mechanism of the ultrahigh frequency of viable hybrid occurrence from lethal crosses not only for plant breeding, which needs to overcome hybrid lethality, but also for understanding the evolution of species. Viable hybrids appeared at a high frequency in reciprocal crosses between *N. amplexicaulis* and *N. tabacum*, suggesting that the cause of viable hybrid occurrence was within the nuclear genome and not in the cytoplasm. Crossing experiments using the S_0_ and S_1_ plants of *N. amplexicaulis* suggested that the *HLA1* gene was not heterozygous and that some other factor must have contributed to the survival of the hybrids.

In crosses using the S_1_ plants, the occurrence rate of viable hybrids varied among S_1_ plants derived from the same parents; 14.1%–29.8% in the pedigree of No. 1 (8.9%), 16.1%–49.2% in the pedigree of No. 2 (63.2%), 6.2%–34.4% in the pedigree of No. 3 (56.8%), and 3.2%–40.3% in the pedigree of No. 4 (51.2%) (**Supplementary Table 3**). If *N. amplexicaulis* is genetically fixed, the frequency is expected to remain constant between the parents and their progeny. Therefore, although the *HLA1* gene was homozygous, some heterozygosity at other loci in *N. amplexicaulis* might have been involved in the fluctuations in the percentages. Alternatively, the frequency of viable hybrids may be influenced by environmental factors, such as temperature during fertilization or seed formation.

Genetic and epigenetic changes have been reported to occur in early generations of new allopolyploids (Shaked et al., 2001; Madlung et al., 2002; Wu et al., 2015; Li et al., 2019; Mhiri et al., 2019; Qiu et al., 2020). Such changes are also observed in interspecific hybrids. Wu et al. (2015) reported that genetic changes ranging from 0.7% to 6.9% and DNA methylation changes ranging from 0.2% to 5.8% were detected in interspecific F_1_ hybrids of *Oryza* using AFLP and MSAP analyses, respectively. In *Nicotiana*, RAPD analysis did not show genetic changes in F_1_ hybrids from several interspecific crosses between *Suaveolentes* species and *N. tabacum* (Tezuka and Marubashi, 2006b; Tezuka et al., 2010; Iizuka et al., 2012; Nakata et al., 2021). However, in the present study, RAPD and AFLP analyses indicated that genomic alterations occurred in the hybrids of *N. amplexicaulis* and *N. tabacum* (**Figure 2**, **Table 2**). In addition, MSAP revealed methylation changes in the hybrids (**Figure 3**). Because genetic and epigenetic changes common to the most viable or lethal hybrids were not detected, it was unclear whether these changes were related to the occurrence of viable hybrids. Nevertheless, the results demonstrated that large-scale genetic and epigenetic changes, commonly referred to as “genome shock” (McClintock, 1984), occurred in interspecific hybrids between *N. amplexicaulis* and *N. tabacum*, suggesting their involvements in ultrahigh frequency appearance of viable hybrids.

We investigated the potential involvement of mutations in *HLA1* and *HLA2*, which are causal genes of hybrid lethality. No mutations were detected at the *HLA2* locus or in the surrounding region in any of the (**Figure 4**). Expression of the *Hla2–1* allele was also observed in all the hybrids (**Supplementary Figure 1**). In contrast, several DNA markers linked to *HLA1* were not detected in eight of the ten viable hybrids, suggesting that this region could be frequently deleted (**Figure 4**). Therefore, one of the mechanisms underlying the ultrahigh-frequency appearance of viable hybrids is the partial deletion of the chromosome where the *HLA1* locus resides.

Crossing results using the S_1_ plants provide insight into the timing of the partial deletion of the chromosome including the *HLA1* locus. If the deletion of *HLA1* occurred during gametogenesis in *N. amplexicaulis*, some S_1_ plants would produce either only viable hybrids (homozygous for the deletion of *HLA1*) or viable and lethal hybrids in a 1:1 ratio (heterozygous for the deletion of *HLA1*). Among the 40 S_1_ plants tested, eight plants produced viable and lethal hybrids in a ratio close to 1:1 (**Supplementary Table 3**, p > 0.05), indicating that the deletion frequency would be 10% (8 out of 80 gametes) if deletion occurred during gametogenesis. However, the overall frequency of viable hybrids from all S_1_ plants was 23.31% (597 out of 2561 F_1_ hybrids). This discrepancy suggests that the deletion of *HLA1* occurs after fertilization rather than during gametogenesis in *N. amplexicaulis*.

Regarding the mechanism of deletion of *HLA1*, if the deletion were produced by reciprocal translocations between homoeologous chromosomes in *N. amplexicaulis* as reported by Nakata et al. (2021), either of the terminal markers on the chromosome carrying *HLA1* would be lost. However, both terminal markers on Nbe.v1.1.chr15 were detected in all viable hybrids (**Figure 4**). Therefore, these hybrids appear to possess a mechanism for eliminating *HLA1* that does not involve reciprocal translocation. Uniparental chromosome elimination following interspecific fertilization has been reported in several plant hybrids (Komeda et al., 2007; Ishii et al., 2010; Sanei et al., 2011). In the cross *Hordeum vulgare* × *H. bulbosum*, inactivation of *H. bulbosum* centromeres triggers uniparental chromosome elimination in the hybrid embryo (Sanei et al., 2011). However, to our knowledge, the mechanisms of large interstitial chromosomal deletions during hybrid formation have not been reported. Our findings suggest that hybrids of *N. amplexicaulis* and *N. tabacum* may possess a novel mechanism for inducing interstitial chromosome deletion through genome shock-induced changes.

In two of the ten viable hybrids examined, deletions at neither causal loci, *HLA1* nor *HLA2*, were detected (**Figure 4**). Genetic and epigenetic changes, as revealed by the RAPD, AFLP, and MSAP analyses (**Figures 2** and **3**), may be involved in the survival of these hybrids. Alternatively, the suppression of causal gene expression may be considered a contributing factor to their occurrence. Based on *Hla2–1* expression analysis, it is unlikely that suppression of *Hla2–1* expression contributed to the occurrence of the two viable hybrids because *Hla2–1* was expressed in all 21 viable hybrids investigated (**Supplementary Figure 1**). As *HLA1* gene has not yet been identified, the expression level of *Hla1–1* could not be assessed in the present study.

It is still unclear whether the epigenetic changes detected by MSAP analysis were linked to differences in gene expression or phenotypic variation among the hybrids. A future transcriptomic analysis, such as RNA-Seq of parental species, viable hybrids and lethal hybrids, could provide comprehensive insights into the molecular basis of hybrid lethality and its suppression. This analysis would help identify pathways downstream of the *HLA1–HLA2* interaction and reveal how the loss of *HLA1* alters gene regulation and restores hybrid viability in *Nicotiana*.

### 4.3 Evolutionary implications of ultrahigh frequency appearance of viable hybrids

Species are generally reproductively isolated from each other. However, the ability of species to overcome reproductive barriers and produce hybrid progenies is evolutionarily intriguing. We showed a phenomenon that enables many hybrids to overcome hybrid lethality and survive in lethal crosses. The section *Suaveolentes* is thought to have originated from the hybridization of a common ancestor of sections *Alatae* and *Sylvestres* with a common ancestor of sections *Noctiflorae* and *Petunioides* (D’Andrea et al., 2023). Considering that most *Suaveolentes* species, including *N. amplexicaulis*, carry the *Hla1–1* allele, *N. amplexicaulis* likely independently acquired the ability to produce a large number of viable hybrids in lethal crosses with *N. tabacum* within the section *Suaveolentes*.

*Suaveolentes* species, which are mainly distributed in Australasia, and *N. tabacum*, which is believed to have originated in South America, are geographically isolated. Although there seems to be no opportunity for these species to intercross, the present study provides a model where sympatric species can overcome hybrid lethality through the ultrahigh-frequency appearance of viable hybrids. When crossed with *N. tabacum*, *Suaveolentes* species other than *N. amplexicaulis* rarely produce viable hybrids, while *N. amplexicaulis* frequently produce viable hybrids. Viable hybrids obtained from crosses between *Suaveolentes* species and *N. tabacum* are generally sterile, and fertility can be restored via amphiploidization through chromosome doubling (Lloyd, 1975; Tezuka and Marubashi, 2006b; Tezuka et al., 2010). In the present study, the viable hybrids obtained from the cross between *N. amplexicaulis* and *N. tabacum* were sterile. Given that speciation requires overcoming both hybrid lethality and hybrid sterility, the ultrahigh-frequency appearance of viable hybrids observed in *N. amplexicaulis* crosses provides a higher chance of overcoming hybrid sterility than crosses that rarely produce viable hybrids.

In plants, many new species are thought to have arisen through interspecific hybridization and whole-genome duplication, with genome shock serving as a driving force that rapidly generates diverse genetic combinations. For instance, field experiments using synthetic interspecific hybrids of the genus *Helianthus* have suggested that the genetic diversity generated by interspecific hybridization accelerates the pace of adaptive evolution (Mitchell et al., 2019). In *Mimulus*, artificially synthesized triploid hybrids overcome hybrid sterility through whole-genome duplication to produce fertile allohexaploids. While these synthetic lines displayed survival rates that were intermediate between those of their parental species, naturally formed triploid*s* (*M*. × *robertsii*), and allohexaploids (*M. peregrinus*) often exhibited even higher survival rates, suggesting that further adaptive evolution under natural conditions can enhance the fitness of emerging polyploid lineages (Meeus et al., 2020). Thus, once a hybrid is established and the factors responsible for reproductive isolation are eliminated, such individuals can acquire novel adaptive capabilities in new environments that are not present in the parental species, leading to a new species.

Future studies could investigate transcriptomic regulation in the viable hybrids obtained from the cross between *N. amplexicaulis* and *N. tabacum* by analyzing expression level dominance (ELD) and homeolog expression bias (HEB), which describe how gene expression patterns are rebalanced between subgenomes following hybridization and polyploidization (Grover et al., 2012; Wu et al., 2018; Li M. et al., 2020). These analyses could reveal how the loss of *HLA1* influences the global regulatory balance between parental subgenomes and whether specific ELD or HEB patterns in stress-related or developmental pathways correlate with the rescue of hybrid viability. This approach would provide broader insights into how interspecific hybridization and structural genomic changes contribute to polyploid genome evolution and phenotypic stabilization.

## 5 Conclusion

In conclusion, although some hybrid seedlings from the cross between *N. amplexicaulis* and *N. tabacum* showed hybrid lethality, others overcame this lethality at ultrahigh frequencies. Our findings indicate that this was primarily due to the elimination of the causal locus *HLA1* for hybrid lethality. Moreover, interspecific hybridization between these species triggers genome shock, which facilitates the breakdown of reproductive barriers, including hybrid lethality. To date, there have been few reports on the mechanisms by which a once reproductively isolated species overcomes these barriers and produced hybrid progeny. The process by which strong hybrid incompatibility is overcome and novel genomic combinations become stabilized not only has significant implications for plant breeding but also serves as a critical model for understanding evolutionary processes in nature.

## Data Availability

The original contributions presented in the study are included in the article/**Supplementary Material**. Further inquiries can be directed to the corresponding author.

## Supplementary material

Supplementary Figure 1 Gene expression of *Hla2–1* in the hybrids from the cross between *N. amplexicaulis* JT and ‘Samsun NN’ analyzed by RT-PCR.

Supplementary Table 1 DNA markers developed in the present study.

Supplementary Table 2 Primers used for AFLP and MSAP analysis.

Supplementary Table 3 Viability of hybrid seedlings between *N. amplexicaulis* JT S_0_/S_1_ plants and *N. tabacum*.

Supplementary Table 4 The number of hybrids lacking specific bands as detected by AFLP analysis.
