# Supplementary material for: Genome-shock deletion of a hybrid lethality gene breaks a reproductive barrier and facilitates speciation in Nicotiana
Source: Front Plant Sci. 2025 Nov 19;16:1690873. doi: 10.3389/fpls.2025.1690873 (PMC12672507; doi:10.3389/fpls.2025.1690873)
Supplement: Supplementary Figure 1 — Gene expression of Hla2–1 in the hybrids from the cross between N. amplexicaulis JT and ‘Samsun NN’ analyzed by RT-PCR. [file Image1.pdf]

Supplementary Material

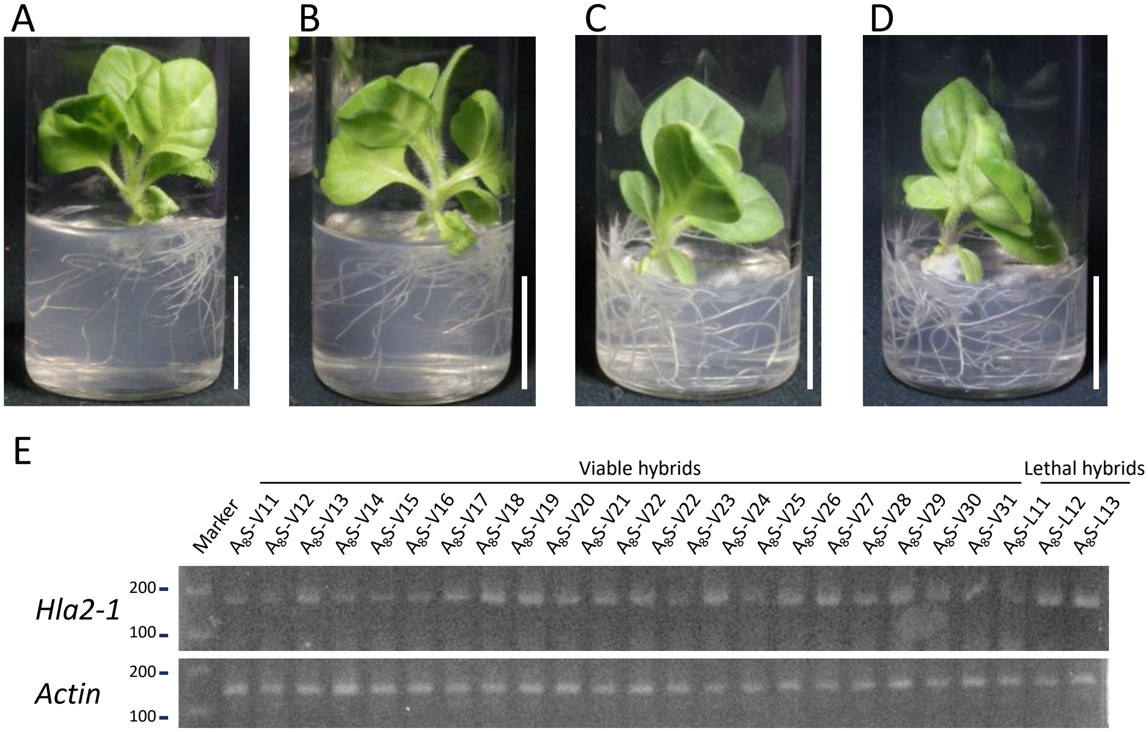

Supplementary Figure S1. Gene expression of *Hla2-1* in the hybrids from the cross between *N. amplexicaulis* JT and 'Samsun NN' analyzed by RT-PCR. (A-D) Hybrids used for RT-PCR analysis. The viable hybrid (A) and the lethal hybrid (C) cultivated at 34°C for 21 days after germination did not show lethality (B) and show lethality (D) after transfer to 28°C for 24 hours, respectively. Scale bars = 2 cm. (E) Electrophoresis of the RT-PCR production. Marker lane, size marker (GeneRuler DNA ladder mix).
